# Bicalcium Phosphate as an Asset in Regenerative Therapy

**DOI:** 10.7759/cureus.44079

**Published:** 2023-08-24

**Authors:** Shefali Maheshwari, Tanishka Taori, Pavan Bajaj, Amit Reche

**Affiliations:** 1 Department of Periodontics, Sharad Pawar Dental College and Hospital, Datta Meghe Institute of Higher Education and Research, Wardha, IND; 2 Department of Public Health Dentistry, Sharad Pawar Dental College and Hospital, Datta Meghe Institute of Higher Education and Research, Wardha, IND

**Keywords:** beta-tricalcium phosphate, bicalcium phosphate, hydroxyapetite, alloplastic bone grafts, bone grafts

## Abstract

After a loss of a tooth, alveolar bone resorption is immutable, leaving the area devoid of sufficient bone quality and mass for a successful and satisfactory implant or any other dental treatment. To treat this problem of irreversible bone loss, bone grafting is the primary solution and a well-accepted technique. The use of bone grafting procedures has increased in recent years. This review is about the various bone grafting techniques and best-situated material available currently along with their trump cards and limitations. In the thorough discussion regarding bone grafting materials and their substitutes, one alloplastic material has shown unbeaten and the most satisfactory properties than any other material, “bicalcium phosphate” (BCP). BCP is a mixture of hydroxyapatite (HA) and beta-tricalcium phosphate (B-TCP) usually obtained through sintering calcium-deficient apatite (CDA) at or above 700°C or by other methods such as hydrolysis or precipitation. The review also shows comparative studies done to understand the effect, most adequate balance, and impact of ratios of HA/B-TCP on the properties, structure, and success rate of this material. The objective of the review is to enlighten the principal characteristic of the most likely used bone graft material presently, i.e., BCP. The most impeccable characteristic of BCP is its capability to osteointegrate, which results in a superior interface. This interface depicts a dynamic process that includes physicochemical reactions, crystal-protein interactions, cell and tissue colonization, and bone remodeling. BCP has certain essential properties that could be put forth as its advantage over any other substitute. These properties include bioactivity, osteointegration, osteoinduction, osteogenesis, and biodegradation, which are mostly governed by modifying the HA/B-TCP ratio. Other applications of BCP are feasible, such as in drug administration and scaffolds for tissue engineering.

## Introduction and background

**Figure 1:**
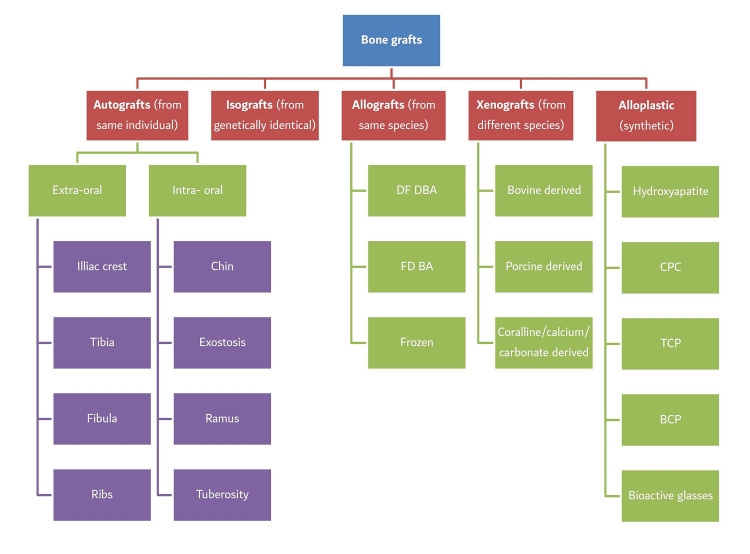
Classification of Bone Graft Materials DF DBA: demineralized freeze-dried bone allograft, FD BA: freeze-dried bone allograft, CPC: calcium phosphate cement, TCP: tricalcium phosphate, BCP: bicalcium phosphate This figure is created by the authors. Sources: [1-4]

Osseous defects are a cavity, indentations, or defects in the alveolar bone involving one or more than one tooth. Bone deformity resulting from surgery, trauma, congenital malformations, or disease is a noteworthy health problem worldwide, which may require bone grafts [1-4]. Various options available to deal with the osseous deformity include increased bone resection, translation of components, bone cement, metal wedges, augmented femoral/tibial components, and bone grafts. Among all, bone grafting has been one of the most stereotyped surgical methods used to augment bone, which is the second most widely used grafting procedure following blood transfusion. A bone graft is described as a viable tissue that, when transplanted into a bony defect alone or in conjunction with other materials, has the ability to induce bone healing. Due to the advances in dental sciences such as implantology, the use of bone grafts or their alternatives in dentistry has expanded dramatically in recent years. Bone tissue has an inherent ability to self-repair, which entails a well-controlled process that restores structure and function in a sequential manner. However, after tooth loss, the alveolar process undergoes irreversible remodeling, reducing the height and width of the remaining ridge. Bone transplant substitutes are classified into numerous groups, including autogenous bone, allogeneic bone, xenogenic bone, and alloplastic alternatives (Figure 1). In context to the preceded statement, the present phenomenal quality graft for repairing bone defects is still considered autogenous bone.

The translocation of grafting material from one bodily location to another within the same individual is known as autografts. Autografts possess osteoconductive, osteoinductive, and osteogenic properties with no immunologic reactions, which makes them a gold standard among osseous grafts [5-8]. These grafts can be obtained from extraoral sites such as the iliac crest, tibia, fibula, and ribs or intraorally from symphysis, exostoses, mandibular ramus, and maxillary tuberosity. However, these grafts have certain limitations, such as an unexplained degeneration pace and the demand for a secondary surgical site, which elevate postsurgical complications among some patients. This compelled the creation of bone substitutes that could combat the constraints of using autogenous bone grafts.

Another grafting technique known as xenograft is also being practiced, which could be bovine-derived, porcine-derived, or coralline calcium-derived [9,10]. Xenograft has osteoconductive properties with minimal limitation and no donor site morbidity [11-13]. However, due to it having no osteogenic and osteoinductive properties, the possibility of disease transmission and ethical problems bounds the use of this grafting technique in certain situations [5].

Another option could be allogenic bone grafts involving grafts from different individuals of the same species, which are available as freeze-dried or demineralized freeze-dried types of bone substitutes [12].

Due to substantial limitations and disadvantages pertaining to autografts and xenografts, the use of another grafting technique known as alloplastic bone grafts and their efficacy in various fields have been studied and researched in recent years [14,15]. Alloplastic bone graft materials can be described as “synthetic,” indicating that they are manufactured from non-organic sources. This material is non-inflammatory and non-carcinogenic, making it safe for patients and highly effective as a bone graft material. These particularly involve bio-glass and polymers. These have full osteoconductive and partial osteoinductive capabilities similar to autografts [16]. Alloplastic bone grafts have now become widely attractive as a viable alternative to autologous bone grafts. When opposed to allogenic and xenogenic bone transplants, alloplastic bone substitutes provide consistent product quality with no risk of infection. Alloplastic bone replacements have several advantages, including biological stability and volume preservation, which allow for cell infiltration and remodeling. The osteoconductive qualities of alloplastic bone substitutes vary depending on their constitutions and production practices, as well as material characteristics, crystal structures, pore sizes, porosities, and absorption rates. These synthetic bone graft substitutes include hydroxyapatite (HA), calcium phosphate cement (CPC), beta-tricalcium phosphate (B-TCP), biphasic calcium phosphate (BCP) (a combination of HA and B-TCP), bioactive glass, and synthetic ceramics [16-21].

## Review

Bicalcium phosphate (BCP)

Of the clinical studies done in the literature demonstrating the preference for BCP over any other grafting material or technique, the capacity to produce a direct bone interaction resulting in a robust junction is the most convincing BCP quality, making it the choice for grafting [6]. Biphasic calcium phosphate is a recently developed bioceramic made by combining hydroxyapatite and beta-tricalcium phosphate. BCP is produced when a synthetic or biological calcium-deficient apatite (CDA) is sintered at a temperature exceeding 700°C [22]. Calcium shortage is determined by different techniques of preparation, such as precipitation, hydrolysis, and mechanical preparation, as well as reaction pH and temperature. This deficient calcium obtained from unsintered apatite determines the proportion of HA and B-TCP to be used. It is observed that the higher the calcium deficiency, the lower the HA/B-TCP fractions [22]. Ample research has been done on the properties and use of varying fractions of HA/B-TCP, the description of which has been depicted in Table 1.

**Table 1:** Uses of Varying Fractions of HA/B-TCP BCP: bicalcium phosphate, HA: hydroxyapatite, B-TCP: beta-tricalcium phosphate This table is created by the authors.

Authors and year	Topic	Ratio of HA (percentage (%))	Ratio of B-TCP (percentage (%))
Yamada et al. (1997) [23]	Comparison of osteoclastic resorption activity of the different compositions of BCP	25	75
Daculsi et al. (1989) [24]	Dogs with periodontal defects treated with implant show healing after six months	85	15
		65	35
		15	85
LeGeros et al. (2003) [22]	To observe the properties of BCP	60	40
Daculsi et al. (1998) [25]	LeGeros eventually found the “tricalcium phosphate” ingredient utilized by Nery, and it was afterward came to known as BCP	80	20
Chen et al. (2014) [26]	To investigate and compare neurovascularization by BCP in various compositions	70	30
		30	70
		2	98
		75	25
Puttini et al. (2019) [27]	Osteoconductive property of BCP	40	60
Cha et al. (2019) [28]	To examine the resorption pattern of BCP, which was utilized to enlarge the maxillary sinuses	70	30
		35	65
		85	15
Lee et al. (2020) [29]	The mechanical properties of BCP-added collagen film and their clinical usefulness in ridge conservation	20	80

Preparation and chemical structure of BCP

Bicalcium phosphate is prepared by different methods, such as sintering, precipitation, hydrolysis, or mechanical preparation. Its chemical reaction is as follows: Ca_10-x_M_x_(PO_4_)_6-y_(HPO_4_)_y_(HO)_2_ -----------------Ca_10_(PO_4_)_6_(HO)_2 _+ Ca_3_(PO_4_)_2_ [6,22].

By hydrolysis, CDA is formulated by the hydrolysis of HCaPO_4_, 2H_2_O in an aqueous solution of NaOH by heating for four hours at 100°C. After that, the solution is filtered and dried for 48 hours at 40°C, with the leftover water being removed by heat treatment [6,22].

By sintering, CDA, either synthetic or biological, is sintered at or above 700°C, forming bicalcium phosphate.

By precipitation, CDA is synthesized by precipitation, followed by simultaneous drops of solutions (Ca (NO_3_)_2_. 4H_2_O and (NH_4_)_2_.HPO_4_) in a three-necked flask at room temperature while the pH is at 11. The powder obtained is thermally heated for one hour to obtain the desired ratio of BCP.

Mechanism of action

**Figure 2:**
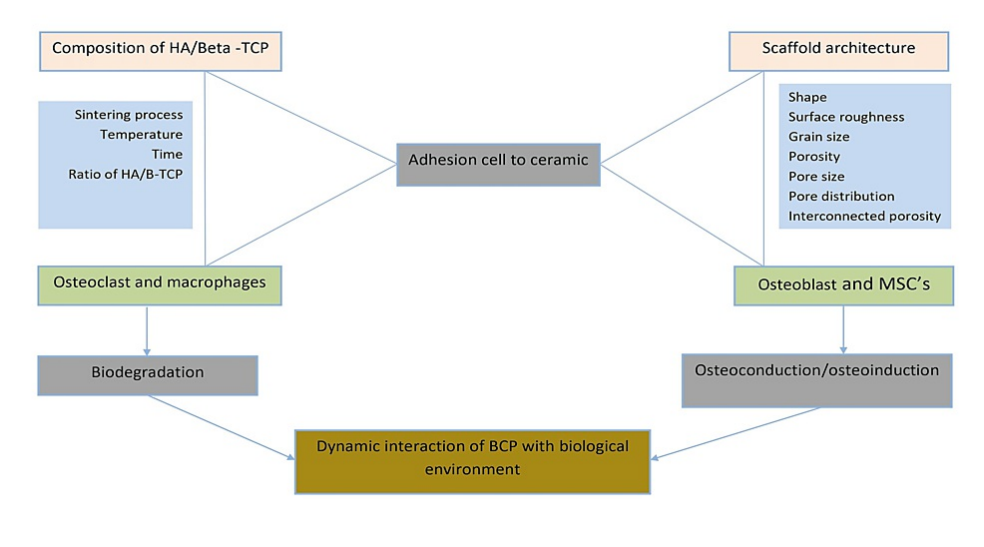
Mechanism of Action of BCP BCP: bicalcium phosphate, MSCs: mesenchymal stem cells, HA: hydroxyapatite, B-TCP: beta-tricalcium phosphate This figure is created by the author. Source: [5]

Figure 2 summarizes the dynamic and complex interaction between the biological environment and biphasic calcium phosphate ceramics. The chemical composition (which depends on the method formation of BCP) and scaffold architecture (including surface roughness and porosity size) of BCP ceramics are eminent criteria for influencing bone cell attachment along with osteoblasts and osteoclasts. The dissolving action and the osteoconduction/osteoinduction capabilities of BCPs are mediated by the auxiliary/associated action of these adhering cells [5,30].

Properties of BCP

Biological Properties

Bone graft materials, such as BCP ceramics, which are bioactive, have the ability and property to build up a direct strong junction with the help of fibrous. Compared to bioinert or bio-tolerant materials, the interface to the host bone creates a strong interface [6]. Equivalent to those elicited by the normal bone, bicalcium phosphate materials also provoke reactions from bone and its related cells in vitro and in vivo. The most important property of osteoinduction and osteogenesis, which are the essential requirement of bone regeneration, is absent in some bioceramics. However, studies done by Reddi show that few bioceramics cause osteoinduction by concentrating the growth factors circulating in the biological fluid at the site of bone augmentation, and these growth factors induce bone regeneration [31]. While a specific bioactive, i.e., BCP ceramic, possesses a ratio of calcium-deficient apatite similar or almost similar to bone apatite crystals, which results in the formation of microcrystals. These microcrystals are essential for BCP’s osteoinductive and osteogenic properties [6,30]. The amount and abundance of the existence of these microcrystals depend upon the relative amount of HA/B-TCP taken during the formulation of BCP. Thus, there is an inverse relation of the proportion of HA/B-TCP with microcrystals formed in BCP, which are essential for the osteoinductive property of this alloplastic material. Hence, the lower the HA/B-TCP, the higher the microcrystal formed and the higher the BCP ceramic’s osteoinductive property [6].

Physical Properties

The mechanical properties of BCP, HA, and TCP have been studied separately numerous times, considering Young’s modulus, compressive strength, flexure strength, Vickers hardness, etc. Various experiments showed the direct proportionality between the ratio Ca/P with elastic modulus and Vickers hardness, i.e., the value of elastic and Vickers hardness increases with an increase in the Ca/P ratio used in the ceramic. The literature has shown the increase in Vickers hardness from 4.9 GPa in TCP to 6.1GPa for HA [32]. Also, Young’s modulus for HA is 122 GPa as against 105 GPa for TCP. Both the above statement infers that as the amount of HA increases in the composite BCP of HA and TCP, the values of Young’s modulus and the Vickers hardness increase. In the context of flexure strength, the strength of TCP proves to be greater than that measured of HA. The maximum flexure strength of BCP ceramic of 202 MPa is achieved when its composition consists of 20% HA [32]. It has also been evidenced that the pore size and the method of preparation of BCP have an influence on the mechanical strength of the bioactive ceramic. A BCP obtained from a single CDA was proposed to have a greater compressive when compared with BCP formed from mixing unsintered calcium phosphate.

Macroporous and Microporous Structures

The average pore size of normal bone is ~ 500 um. It has been demonstrated and studied that the pore size of BPC is 565 um, which is again evidence that BCP could be an ideal bone substitute. Macroporosities having a diameter >100 um provide a framework for bone-cell colonization, whereas microporosities having a diameter of <10 um allow body fluid circulation (which consists of growth factors) and hence help in bone renewal [22]. The size of these micro-/macroporosities is determined by the method/type of manufacturing procedure of BCP ceramics, the incorporation of volatile materials such as naphthalene, hydrogen peroxide, etc., followed by heating below the temperature of 200°C, and subsequent sintering at higher temperatures [22]. This led to the formation of macroporosity in the formed BCP ceramic by the evaporation of volatile substances. Microporosity results from the duration and the temperature of sintering, and as the time and temperature of sintering increase, the porosity also increases. Microporosity has an important function in improving the osteogenic differentiation of bone tissue engineering applications. Microporosity increases the specific surface area, which accelerates the liberation of degradation products by generating more area for protein adsorption. The faster the breakdown ingredient is released, the easier it is for scaffolds and cells to interact. Rouahi et al. have studied the effect of microporous HA on serum protein adsorption [33]. This study demonstrated that microporous HA adsorbs 10 times more proteins than non-microporous HA, including fibronectin and albumin. These microporosities provide a capillary force that encourages bone-related cells to adhere to the scaffold’s surface [33].

Application of BCP

As described before, the alloplastic material bicalcium phosphate (BCP) is a potent bone graft material. The application and utility of BCP are broad spectrum (Table 2).

**Table 2:** Various Applications of BCP BCP: bicalcium phosphate, HA: hydroxyapatite, BCPC: bicalcium phosphate ceramic, CaP: calcium phosphate, PRF: platelet-rich fibrin, B-TCP: beta-tricalcium phosphate, rhBMP-2: recombinant human bone morphogenetic protein-2 This table is created by the author.

Application of BCP
BCP is used as a bone reconstructing and bone graft material [34,35].
Apart from its use in medical and dental professions, BCP has a number of other impending utilities, including antibiotics, growth factor carriers, medication delivery systems, hormones, and tissue engineering scaffolds [6].
BCPC can also be used as a grit-blasting abrasive for surface modification of the implant substrate [22].
BCPC can be applied in maxillary sinus augmentation [28].
Clinically positive ridge preservation findings were reported that the UV cross-linked and BCP- added collagen films have equivalent biocompatibility and mechanical qualities as chemically cross-linked collagen membranes or films [29].
When rhBMP-2 was combined with BCP and BCPC, bone regeneration was greatly accelerated, and during the early phases of healing, BCPC resulted in a complex matrix of new bone nanoparticles. As a result, BCPC is a viable candidate for rhBMP-2 transport [36].
In vivo and in vitro, both types of research have shown evidence of neurovascularization of various porous CaP ceramics (BPC in different ratios). After being experimented by implanting into the thigh muscle of mice, rapid neurovascularization has been observed [26].
BCP is made up of ratios. An investigation in rat calvarial revealed that 60% HA/40% TCP induced novel bone creation by osteoconduction and might be used as a surrogate in bone revival operations [27].
In a technique to evaluate the maxillary sinus floor, BCP was used [37,38].
For the treatment of furcation anomalies, a clinical study trial with autologous PRF in collaboration with HA and B-TCP or HA and B-TCP alone was conducted [39-41].

Advantages

Alternative to Autografts

BCP bioceramics, which have recently been popular, can be used as an alternative to autogenous bone transplants in orthopedic and dental therapies. It is now accessible in the form of particles, blocks, and customized designs for a variety of applications [22,42].

Osteoinductive/Osteogenic Property

This property of BCP ceramic is attributed to the similarity of CDA ratios (essential for the formation of microcrystals) in ceramic to those of bone apatite crystals observed after implantation of MBCP [11]. The four paramount properties of osteoinduction, osseointegration, osteoconduction, and osteogenesis are incorporated in this bioceramic [25,37,43-46].

Biological Stability

TCP has great potential to dissolve readily in the biological setting. Some studies done by authors have shown that this rapidly dissolving nature renders the B-TCP unstable. The advent of biphasic calcium phosphate consisting of a combination of HA (having low dissolution property) and B-TCP (with more dissolution property) make this ceramic a better biodegradable and comparatively stable in the biological environment [44,47].

Bioactive Concept

BCP is composed of a balanced blending of highly stable HA and TCP, which is highly soluble. This alloplastic material rapidly biodegrades in a biological medium, resulting in new bone generation by the release of calcium and phosphate ions. This property of bioactivity and its fast biodegradation can be regulated by varying the HA and B-TCP percentage [25,35,47,48].

Direct Osteointegration

The capacity of BCP to produce direct bone bonding, which leads to a robust interface, is its most enticing feature. This interface necessitates a series of interactions with cells, as well as dissolution and precipitation processes.

Superior Properties Over Either of the Components

Various studies provided evidence that the TCP has faster biodegradability and absorption compared with HA, while HA has better biostability and physical properties as compared to TCP. Hence, integrating both alloplastic materials in BCP makes BCP the most acceptable alloplastic material compared to pure forms or any other synthetic bone graft substitutes developed so far. Clinical studies have revealed that interleukins and minor posterior bone transplants benefit bone regeneration and periodontitis treatment [49,50].

**Figure 3:**
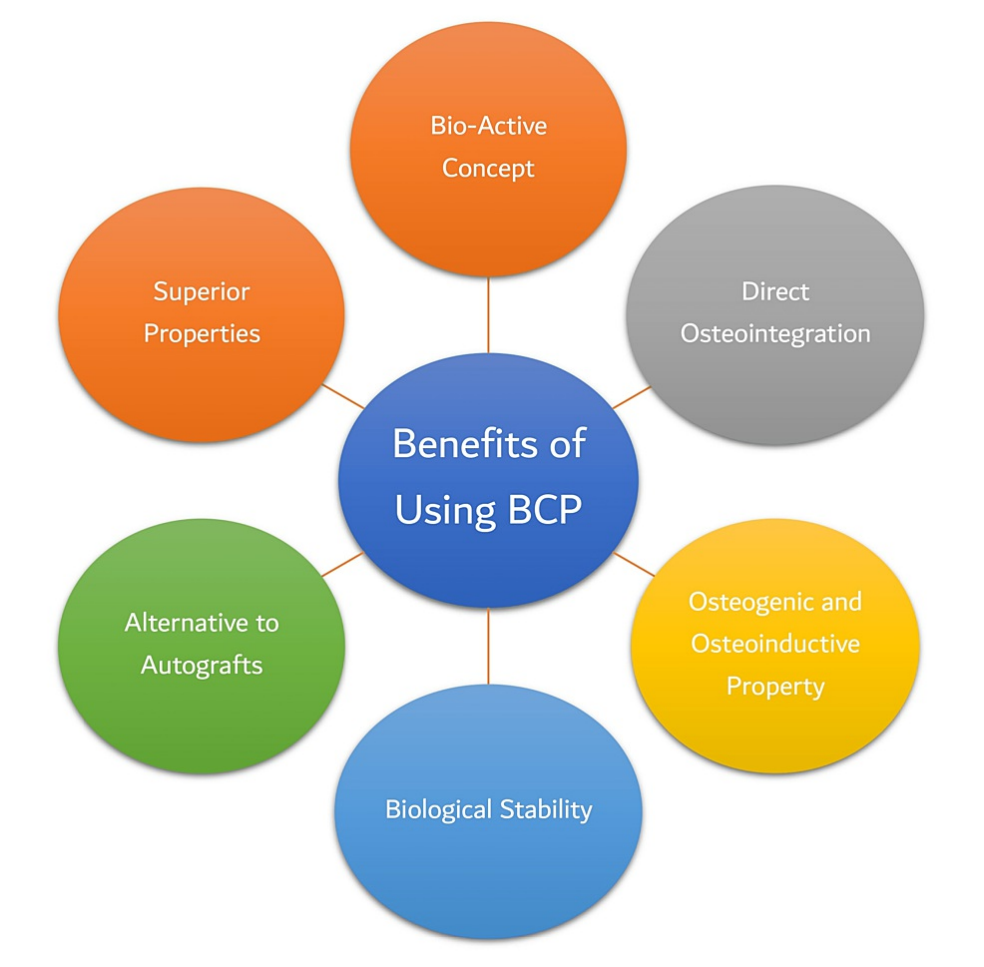
Various Uses of BCP BCP: bicalcium phosphate This figure is created by the author. Source: [25,37,43-47,49,50]

Figure 3 summarizes the various uses of BCP.

## Conclusions

New techniques and methods have been evolving to replace the human body’s missing, traumatized, or defective hard structures. Bone grafts and various bone substitute materials, in the form of nanoparticles or blocks, have been used in dental practices as a substitute for human bone. With the increasing quest to overcome this bone defect, an advanced alloplastic bone substitute was introduced, which is the combination of hydroxyapatite (HA) and beta-tricalcium phosphate (B-TCP), i.e., bicalcium phosphate ceramic (BCP). This complex mixture of two different synthetic bone substitutes leads to the formation of comparatively even better bone graft substitutes (BCP) having superior properties. Due to bioactive, biocompatibility, biodegradable, osteoconductive/osteogenesis, and osteointegration properties and resemblance to natural bone, BCP, a synthetic substitute, has become a propitious and encouraging replacement for autologous and other bone grafting techniques. This bone substitute may be used because of its reduced risk, required resorption rate, and ability to be employed with growth hormones or cell transplantation. The varying percentage contribution of HA and B-TCP in BCP greatly influences the abovementioned physical and biological properties and surface structure (macro-/microporosity). The period of healing and the amount of donor material used has an impact on BCP dissolution. This review also compiles the interplay between the biological and mechanical properties of the microporous framework. According to studies, the essential mechanical attributes and the mechanical qualities of a matrix used in bone tissue engineering applications are influenced by its homogeneity. Despite the benefits and impeccable properties, more research is needed to understand and develop new dental biomaterials with controlled degradation, structural and mechanical stability, and remodeling ability that approximates the rate of new bone production, as highlighted in this review paper. Although numerous types of research have been done on BCP for its innumerable application, still, in vivo research is required to recognize and create the optimum needed HA and B-TCP percentage distribution and the ideal porosity of BCP-based bone replacements. Alloplastic bone with excellent safety and uniform quality may become the preferred choice in the treatment of skeletal abnormalities and bone augmentation in the near future.
